# Characterisation of the Introgression of *Brassica villosa* Genome Into Broccoli to Enhance Methionine-Derived Glucosinolates and Associated Health Benefits

**DOI:** 10.3389/fpls.2022.855707

**Published:** 2022-04-01

**Authors:** Mikhaela Neequaye, Burkhard Steuernagel, Shikha Saha, Martin Trick, Perla Troncoso-Rey, Frans van den Bosch, Maria H. Traka, Lars Østergaard, Richard Mithen

**Affiliations:** ^1^Quadram Institute Bioscience, Norwich, United Kingdom; ^2^John Innes Centre, Norwich, United Kingdom; ^3^Bayer, Wageningen, Netherlands; ^4^Liggins Institute, University of Auckland, Auckland, New Zealand

**Keywords:** broccoli, *Brassica oleracea*, glucoraphanin, transcription factor, transcriptome, introgression, sulphur metabolism, *MYB28*

## Abstract

Broccoli cultivars that have enhanced accumulation of methionine-derived glucosinolates have been developed through the introgression of a novel allele of the *MYB28* transcription factor from the wild species *Brassica villosa*. Through a novel k-mer approach, we characterised the extent of the introgression of unique *B. villosa* genome sequences into high glucosinolate broccoli genotypes. RNAseq analyses indicated that the introgression of the *B. villosa MYB28* C2 allele resulted in the enhanced expression of the *MYB28* transcription factor, and modified expression of genes associated with sulphate absorption and reduction, and methionine and glucosinolate biosynthesis when compared to standard broccoli. A adenine-thymine (AT) short tandem repeat (STR) was identified within the 5′ untranslated region (UTR) *B. villosa MYB28* allele that was absent from two divergent cultivated forms of *Brassica oleracea*, which may underpin the enhanced expression of *B. villosa MYB28*.

## Introduction

The *Brassica oleracea*, *n* = 9, species complex comprises several agronomic varieties and a series of partially sexually compatible wild perennial “species” that are found mainly on Mediterranean coastal cliff faces and associated habitats (Lanner et al., 1997). Genome sequence analyses suggest that these wild species may represent a source of novel genes and alleles for breeding of *B. oleracea* and also the amphidiploid *Brassica napus* (*B. napus*) (Golicz et al., 2016). Despite the attraction of new sources of genetic diversity, the use of wild species of the *B. oleracea* complex is challenging due to the time taken to recover agronomically acceptable phenotypes and potentially deleterious effects of non-targeted introgression, a limitation common to the use of wild species in all crop development programmes.

Oilseed and horticultural *Brassica* crops accumulate sulphur-containing glycosides commonly known as glucosinolates in vegetative and reproductive tissues. The glucosinolate molecule comprises a common sulphated aldoxime with a variable side chain derived from a small number of amino acids. Following tissue disruption, glucosinolates are hydrolysed to an array of bioactive compounds. Many of these hydrolytic products have been shown to mediate plant-herbivore and plant-pathogen interactions. The hydrolytic products of 2-hydroxy-3-butenyl glucosinolate that accumulates in the seeds of oilseed brassicas have goitrogenic activity when the protein-rich meal is fed to cattle, which led to the breeding and widespread adoption of low seed glucosinolate cultivars. Certain glucosinolates within horticultural brassicas, and in particular, 4-methylsulphinylbutyl glucosinolate (“glucoraphanin”) and 3-methylsulphinylpropyl glucosinolate (glucoiberin) derived from methionine and which accumulate in *B. oleracea* var. *Italica* (broccoli), have been associated with the putative health-promoting properties of cruciferous vegetables.

Wild forms of *B. oleracea* have been used to explore the genetic basis of the chemical structure of the glucosinolate side chains. A small set of Mendelian genes interact to determine the length and chemical structure of the glucosinolate side chains. *B. villosa*, a wild form of *B. oleracea* found in Sicily, has been used as a source of a “high glucoraphanin” trait for broccoli breeding programmes for use in studies to explore the health-promoting activities of broccoli (Armah et al., 2013; Armah et al., 2015) and the manner by which high glucoraphanin broccoli may reduce the risk of aggressive prostate cancer (Traka et al., 2019), as described below.

The development and phenotypic characterisation of *B. villosa*-derived high glucoraphanin broccoli (trademarked as Beneforte) have been previously described, and low-density KASpar markers were used to identify regions of genomic introgression of *B. villosa* into the broccoli genetic background (Traka et al., 2013). These F_1_ hybrids were shown to contain a *B. villosa* allele of the *MYB28* transcription factor on chromosome 2 resulting in greater expression of *MYB28* in these hybrids than standard broccoli hybrids (Traka et al., 2013). Complementary studies in both *Arabidopsis* and *Brassica* have shown that enhanced *MYB28* expression results in the greater accumulation of methionine-derived glucosinolates and identified *MYB28* as the major transcriptional regulator of aliphatic glucosinolate biosynthesis (Hirai et al., 2007; Sonderby et al., 2010; Seo et al., 2016; Yin et al., 2017).

A 12-month dietary intervention study with men who had organ-confined prostate cancer demonstrated a dose-dependent association between the *MYB28 B. villosa* allele (*MYB28* V) and the suppression of changes in oncogenic gene expression in prostate biopsy tissue. Men who consumed a broccoli soup manufactured from the standard broccoli cultivar Ironman (genotype *MYB28* B/B) exhibited changes in gene expression in prostate tissue consistent with enhanced cancer risk, which was partially suppressed with Beneforte (*MYB28* B/V) and entirely suppressed with a non-commercial broccoli genotype high-glucoraphanin (HG) Inbred, which was homozygous for the *B. villosa* allele (*MYB28* V/V) (Traka et al., 2019).

Whilst it was assumed that the high glucosinolate trait and the suppression of changes in gene expression in prostate tissue were due to the introgression of the *MYB28* V allele, it is conceivable that additional non-target introgressed genomic segments may have been of importance, particularly, in the HG Inbred genotype that completely suppressed changes in oncogenic gene expression. Moreover, the role of the additional two paralogues of *MYB28*, on chromosomes C7 and C9, in contributing to the high glucosinolate trait has not been determined. In the current study, we quantify through genomic sequencing and K-mer analyses the extent and distribution of the introgression of the *B. villosa* genome into HG Inbred and the agronomically superior high glucoraphanin F_1_ broccoli hybrid 1086, both of which are homozygous for the *B. villosa MYB28* C2 allele. Furthermore, through RNAseq, we seek to investigate the consequences of the enhanced expression of *MYB28* on gene expression associated with glucosinolate biosynthesis. Lastly, we speculated as to a possible regulatory element that results in enhanced expression of the *B. villosa* allele.

## Materials and Methods

### Plant Material

Four broccoli genotypes were used within the research project: (1) Ironman, a widely grown commercial F_1_ hybrid broccoli that is homozygous for the *MYB28* C2 broccoli allele (B/B). (2) 1199 (tradename Beneforte), an F_1_ hybrid that is heterozygous for the *MYB28* C2 *B. villosa/*broccoli allele (V/B). (3) HG Inbred, an inbred genotype that is homozygous for the *B. villosa MYB28* C2 allele (V/V). (4) 1086, an elite broccoli F_1_ hybrid that is homozygous for the *MYB28* C2 *B. villosa* allele (V/V). Whilst both 1086 and HG Inbred have the same *MYB28* C2 genotype (V/V) but the genetic background is different with 1086 exhibiting an elite broccoli agronomic phenotype in contrast to HG Inbred. These four genotypes were grown under normal agronomic conditions in an experimental field plot in Norwich in 2017. Leaves were sampled from three plants of each genotype for genome sequencing and gene expression analyses, and florets were sampled for metabolite analyses. All leaf material was harvested whilst the plants were in a vegetative stage prior to the emergence of inflorescences. Additional leaves were sampled from a single glasshouse-grown *B. villosa* plant for genomic and gene expression analyses.

### Genome Sequencing Analysis

DNA was isolated from leaf material using a phenol-chloroform extraction protocol (Kang and Yang, 2004). Genomic DNA libraries were prepped and sequenced using Illumina HiSeq paired-end sequencing with 30X coverage by Novogene©^1^ producing an average of 125 million total reads per sample with 35X average depth coverage (Supplementary Table 1). The total length of assemblies varied between 396,236,782 and 427,100,513 bp, with the number of contigs ranging from 108,592 to 195,078 (Supplementary Table 2). Raw data from each line were trimmed using Trimmomatic version 0.33 (Bolger et al., 2014) with parameters LEADING:20 TRAILING:20 SLIDINGWINDOW:10:20 MINLEN:50. Reads were assembled into contigs using CLC Assembly Cell^2^ with default parameters.

### Anchoring and Visualisation

Contigs from the CLC draft assemblies were anchored using gene models of the reference genome of *B. oleracea* TO1000 (Assembly BOL, INSDC Assembly GCA_000695525.1 version 97.1) (Parkin et al., 2014). Coding sequences (CDS) of each gene model were aligned to an assembly using NCBI BLASTN (Chen et al., 2015) with default parameters. For each gene model, the contig with the highest alignment score was selected and the genomic position of the gene model was assigned to the contig. Plots were visualised and generated using RStudio v.1.0.143 (Racine, 2012).

### Introgression Analysis

K-mers were counted in raw-data of *B. villosa* (donor) and Ironman (background) using jellyfish^3^ version 2.1.4. (Marçais and Kingsford, 2011). The parameter “-C” was used for jellyfish to handle a k-mer along with its reverse complement as one item. Sequence data from the draft assembly of the introgression line and k-mer presence or absence within the donor and background were used to determine the ratio of k-mers that are shared between donor and introgression line vs. the ratio of k-mer that is shared between background and introgression line. A custom java programme calculated the ratio of k-mers between the “reference” high glucoraphanin assembly and the “wildtype” (Ironman) and “donor” (*B. villosa*) per contig. For each contig that was able to be anchored to the TO1000 *B. oleracea* assembly (Parkin et al., 2014), this ratio was visualised. For each contig of a CLC assembly, the set of k-mers (*k* = 31) was recorded. Subsequently, it was determined how many of those k-mers were present only in the raw data of the “donor” *B. villosa* and how many were present only in the raw data of the commercial broccoli background, Ironman. The resulting plot displays the ratio of k-mer alignment between these lines in which k-mer alignment with the *B. villosa* appears in black.

### Gene Expression Analysis

Transcriptome analysis was undertaken on vegetative leaves from field-grown individual plants of field-grown Ironman and HG Inbred in addition to a glasshouse-grown *B. villosa* leaf of similar developmental stage. In *Arabidopsis*, leaves are the site of the biosynthesis of aliphatic glucosinolate that is subsequently transported to roots and reproductive tissues (Andersen and Halkier, 2014; Madsen et al., 2014; Jorgensen et al., 2015). Likewise, in broccoli, leaf expression of *MYB28* correlates with the expression of glucosinolates in florets as opposed to the expression of *MYB28* in florets themselves (Traka et al., 2013). RNA extraction was performed using E.Z.N.A.^®^ Plant RNA Kit provided by Omega Bio-tek Inc. A total of seven individual plant RNA samples (three Ironman, three HG Inbred, and one *B. villosa*) were used to generate TruSeq non-directional RNA libraries at Earlham Institute (United Kingdom). Libraries were sequenced in one pool of seven (7-plex) and run on two lanes of the Illumina HiSeq2500 with a 125 bp paired-end read metric that generated an average of 160,170 million reads per sample. Sequencing was performed by the Earlham Institute. Individual plant sample RNA was used to generate 7 TruSeq non-directional RNA libraries. These libraries were sequenced through pooling in one pool of 7 (7-plex) and run on 2 lanes of the Illumina HiSeq2500 with a 125 bp paired-end read metric with an average of 160–170 million reads per sample. This data are available on NCBI under Project ID PRJNA623495.

Data analysis of RNA-seq raw data was conducted following the protocol for the Tuxedo suite for short reads with default parameters (HISAT2, v.2.0.4, StringTie, v.1.2.2, Ballgown, v.2.8.4) (Pertea et al., 2016). Differential expression analysis was performed using the Ballgown package in RStudio (Racine, 2012; R Core Team, 2013). An enrichment analysis was conducted on the list of differentially expressed (*p* < 0.05) genes that was generated using the Ballgown software included in the “Tuxedo Suite” for RNA-seq data analysis (R Core Team, 2013), which provided the significantly differentially expressed genes in the HG Inbred *MYB28* V/V broccoli when compared to standard broccoli cultivar Ironman *MYB28* B/B (*p* < 0.05). This data set was processed using TopGo analysis to gain a list of enriched gene ontologies in the dataset (Alexa and Rahnenführer, 2009). The gene set analysis statistically compared the representation of Gene Ontology (GO) terms, which were first assigned to the *B. oleracea* gene models using the *Arabidopsis thaliana* org.At.tair.db library (version 3.2.3) (Carlson, 2016), in this gene set to that of the “expected” value to determine those to be considered “over-represented” using the BP parameter with a node size of 10, then confirming the statistical significance of this using Fisher’s exact test. This was performed in RStudio v.1.0.143 (Racine, 2012). A Multidimensional Scaling (MDS) of Fragments Per Kilobase Million (FPKMs) displaying Euclidean distances between these samples was analysed as a “principal component” analysis to address the comparison of clustering between replicates of the different genotypes. This was performed using gene expression data, following the removal of low abundance transcripts. Independent comparative analyses included *t*-tests of Transcripts per Kilobase Million (TPM) in the RNA TPM in the RNA-seq analysis when comparing expression in the standard broccoli with the HG Inbred (Figure 3).

Gene expression analyses of three genes, *APS3* (Bo5g021810), *MAM* C7 (Bo7g098000), and *CYP79F1/F2* (Bo5g021810), were additionally quantified by quantitative reverse transcription PCR (RT-qPCR). *MAM* C7 has previously been referred to as *MAM3* (Yin et al., 2017). Primer sequences can be found in Supplementary Table 3. RT-qPCR amplification was from the RNA extractions that had generated cDNA using the REVERSE TRANSCRIPTASE M-MLV Kit Supplied by Life Technologies Ltd. Reactions included 5 μl RNA, 1 μl oligo dT, 1 μl deoxynucleotide 5′-triphosphates (dNTPs), and 5 μl distilled H2O and were run at 65°C for 5 min. This was followed by the addition of 4 μl of 5 × buffer and 2 μl 0.1 M Dithiothreitol (DTT) before being kept at 37°C for 2 min. Finally, 1 μl Moloney Murine Leukemia Virus (MMLV) reverse transcriptase was added to the mixture before being incubated at 37°C for 50 min followed by 70°C for 15 min. Concentrations of 150 ng/μl of cDNA were used for RT-qPCR reactions. Gene expression was quantified using the QuantiNova SYBR Green PCR Kit from Qiagen. PCRs were carried out in a Bio-Rad CFX96 machine (C1000 Touch). The PCR cycling conditions were 95°C for 15 min, 40 cycles of 95°C for 15 s, and 60°C for 60 s.

### Metabolite Analysis

Sulphate and S-methylcysteine sulfoxide (SMCSO) analyses were performed as described previously with minor modifications (Koprivova et al., 2008; Kubec and Dadáková, 2009). Glucosinolate analysis was performed as previously described (Saha et al., 2012). Freeze-dried inflorescences resembling commercial edible broccoli floret samples were sent to the Eurofins Food and Feed Testing Laboratories for quantification of total sulphur, cysteine, and methionine by high-performance liquid chromatography (HPLC) analysis. Phenotypic analyses of metabolites included a one-way ANOVA comparing metabolite content of each of the broccoli cultivars for a single year along with Tukey’s multiple comparison tests. All statistical analyses were carried out on GraphPad Prism (version 8.2.0).

## Results

### Metabolite Analyses

**FIGURE 1:**
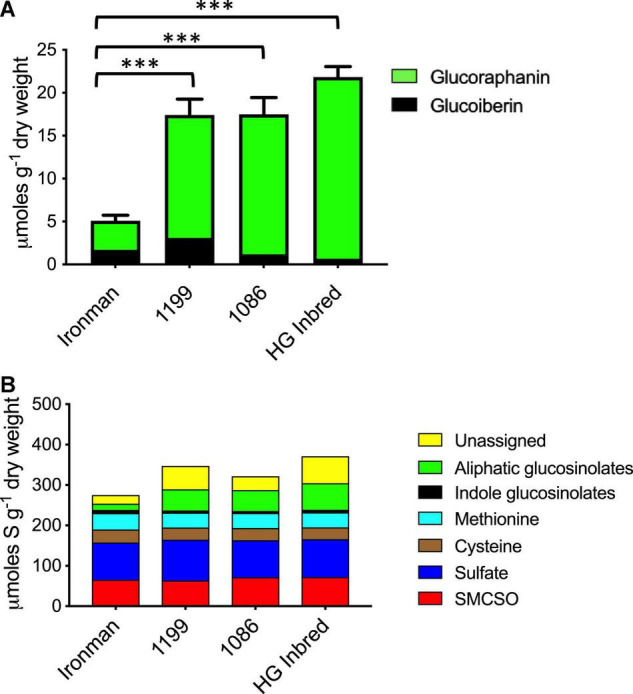
**(A)** Aliphatic (methionine-derived) glucosinolates in the standard broccoli F_1_ hybrid Ironman and in three high glucoraphanin broccoli genotypes, 1199, 1086, and high-glucoraphanin (HG). Ironman has significantly fewer glucosinolates than each of the three high glucoraphanin genotypes (^***^p ≤ 0.0001; Tukey’s multiple comparison test details in Supplementary Table 3). **(B)** Partitioning of sulphur within the major sulphur-containing metabolites in Ironman, 1199, 1086, and HG. There are no significant differences (*p* < 0.05) in the total sulphur content or any specific metabolite apart from aliphatic glucosinolates as shown in panel **(A)**.

Field-grown broccoli florets of the four genotypes described above were analysed for their glucosinolate phenotype. Broccoli genotypes that had either one or two *MYB28* V alleles had significantly greater total content of the aliphatic methionine-derived glucosinolates glucoraphanin and glucoiberin in their florets (Figure 1A and Supplementary Table 4), but there was no difference in glucosinolate content between genotypes that were homozygous (HG Inbred and 1086) or heterozygous (1199) for the *MYB28* C2 V allele. There were no significant differences in tryptophan-derived glucosinolates or the other major sulphur-containing broccoli metabolites, S-methylcysteine sulfoxide, methionine, cysteine, and sulphate (Figure 1B).

### *Brassica villosa* Genome Introgression

Three broccoli genotypes (Ironman, HG Inbred, and 1086) and one accession of *B. villosa* were sequenced to ≈35-fold coverage using Illumina PE sequencing. Two of the broccoli genotypes, 1086 and HG Inbred, contained introgressions from *B. villosa* and had high levels of glucoraphanin. The third genotype, the commercial broccoli Ironman, lacked any introgression from *B. villosa* and was used as a partial surrogate for the broccoli genetic background of the genotypes with *B. villosa* introgressions.

**FIGURE 2:**
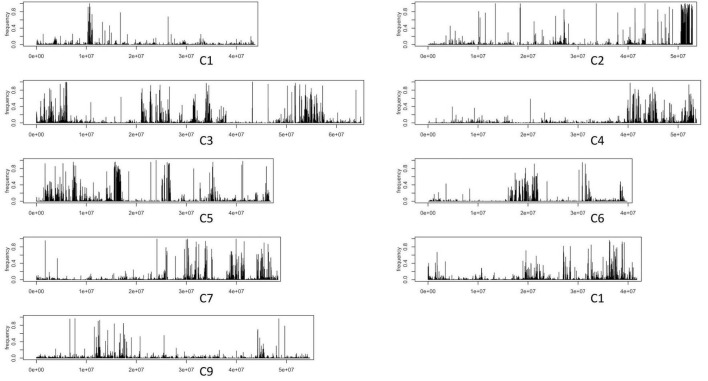
Signals for potential introgression traces from *Brassica villosa* Introgression from k-mer analyses. In the 1086 F_1_ hybrid, each black horizontal line in the plot marks a contig of a draft-assembled high-glucoraphanin genome. The height of each line is according to the fraction of k-mers in a contig that is unique to *B. villosa*.

Draft gene-space assemblies of 1086 and HG Inbred were generated. Gene-containing contigs were anchored to the *B. oleracea* reference assembly of TO1000 (Parkin et al., 2014). For each anchored contig of an assembly, its set of k-mers (*k* = 31) was determined. We additionally obtained all k-mers from the raw data of Ironman and *B. villosa* (Table 1). Testing for each contig of an introgression genotypes assembly involved quantifying how many of its contigs k-mers were unique to *B. villosa* and how many were unique to Ironman. This enabled to ascertain which contigs represented sequence from introgressed *B. villosa* segments. The anchored positions for contigs that likely resembled a signal for true were identified (Figure 2). As was expected, the most pronounced region of introgression was towards the end of C2 where *MYB28* has previously been positioned (Traka et al., 2013), but there was evidence of additional introgression across all other linkage groups. The distribution of introgressed segments of *B. villosa* genome was similar in the elite F_1_ hybrid 1086 to the HG Inbred (Figure 2 and Supplementary Figure 1). It is notable that despite the widespread introgression of *B. villosa* into 1086, this genotype has an elite agronomic phenotype with no apparent negative traits.

**TABLE 1:** Contig anchoring and K-mer analyses of *Brassica villosa* introgression genotypes HG and 1086.

	Number of anchored contigs	Sequence length of anchored contigs	Unique kmers ironman	Unique kmers *B. villosa*
HG Inbred	33,142	201,787,918	40,596,193	910,719
1086	23,508	206,013,069	40,393,853	1,475,642

### Gene Expression Analyses

**FIGURE 3:**
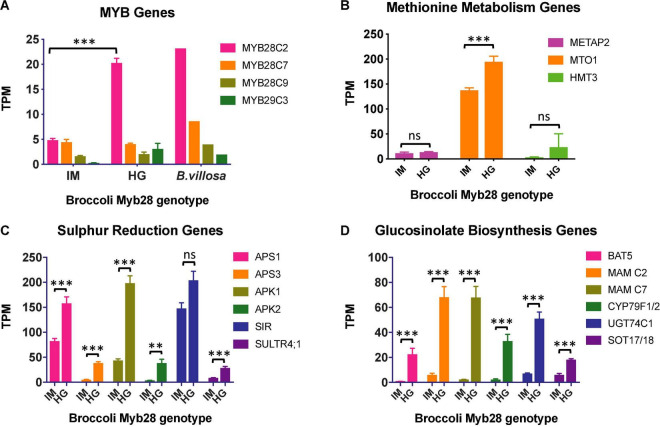
**(A)** Relative expression (mean and se) of genes from RNAseq analyses in leaves from field-grown Ironman (IM) and high-glucoraphanin (HG) Inbred (HG). **(A)** *MYB* transcription factors. There is a significantly greater expression of *MYB28* C2 in HG Inbred than IM (*p* < 0.001). Other *MYB28* C7, *MYB* 28 C9, and *MYB29* C3 genes do not differ significantly in expression between HG and IM (*p* > 0.05). For information, gene expression of *MYB28* genes is also included from a single leaf of glasshouse grown *Brassica villosa*. **(B)** Sulphate reduction. There was significantly greater expression of sulphate reduction genes in HG Inbred compared to IM (^***^p < 0.01; ^**^p < 0.05) with the exception of *SIR* (*p* > 0.05). **(C)** Methionine metabolism. Expression of *MTO1* was greater in HG Inbred compared to IM (^***^p < 0.01). *METAP2* and *HMT3* did not differ significantly between HG Inbred and Ironman. **(D)** Glucosinolate biosynthesis. There was significantly greater expression of all glucosinolate biosynthesis genes in HG Inbred compared to IM (^***^p < 0.01).

The overall percentage alignment of RNAseq reads from the three samples, each of Ironman and HG Inbred to the TO1000 *B. oleracea* reference genome (Parkin et al., 2014), was 90.0 ± 0.31 and 90.7 ± 0.48, respectively. The alignment of *B. villosa* and TO1000 was significantly lower at 82.3% (*p* < 0.001), indicative of its greater divergence from TO1000 when compared to broccoli. 3286 genes were differentially expressed between Ironman and HG Inbred (uncorrected *p* < 0.05). Of the three *MYB28* paralogues on C2, C7, and C9, only the *MYB28* C2 allele was differentially expressed between Ironman and HG Inbred, with levels of expression in HG Inbred similar to that in *B. villosa* (Figure 3A). Examples of relative expression of genes involved in sulphate reduction, methionine metabolism, and glucosinolate biosynthesis are shown in Figures 3B–D, respectively. RT-qPCR independently confirmed the enhanced expression of three genes—*APS3*, *MAM* C7, and *CYP79F1/2*—in HG Inbred when compared to Ironman. Gene ontology analyses confirmed the significant difference of several metabolic pathways associated with sulphur metabolism and glucosinolate biosynthesis (Table 2).

**TABLE 2:** Gene Ontology (GO) terms associated with sulphur metabolism based on enriched genes differentially expressed (*p* < 0.05) between HG *MYB28* V/V broccoli compared to 9 standard broccoli *MYB28* B/B.

GO.ID	Term	Annotated	Number differentially expressed	Expected	*p*
GO:0006534	Cysteine metabolic process	377	33	21.03	0.0073
GO:0042762	Regulation of sulphur metabolic process	36	7	2.01	0.0033
GO:0010439	Regulation of glucosinolate biosynthesis	16	6	0.89	0.0002
GO:0006555	Methionine metabolic process	168	24	9.37	< 0.0001
GO:0000103	Sulphate assimilation	20	8	1.12	< 0.0001
GO:0019760	Glucosinolate metabolic process	368	51	20.53	< 0.0001
GO:0006790	Sulphur compound metabolic process	1232	135	68.72	< 0.0001

### Genomic Analyses of *MYB28* C2

**FIGURE 4:**
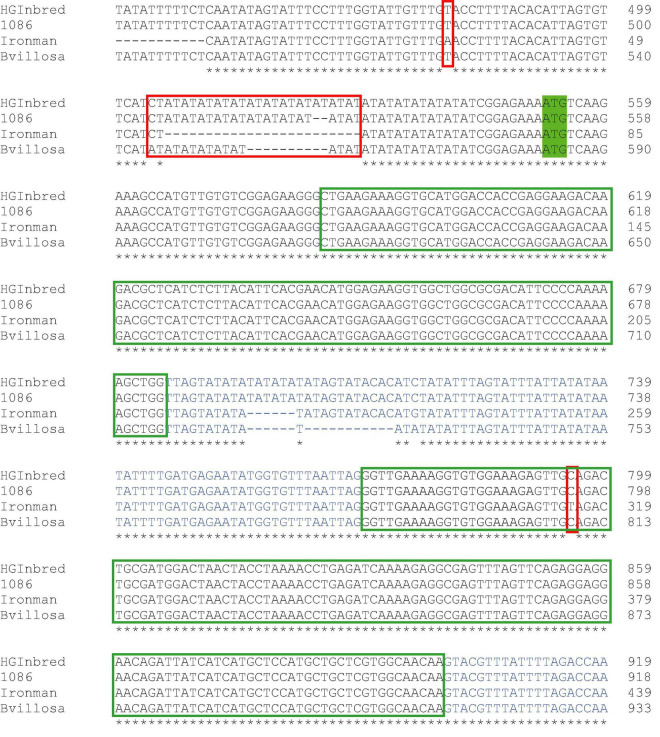
Part of the genomic sequence of the *MYB28* C2 allele from the standard broccoli Ironman, *Brassica villosa*, and the high glucoraphanin broccoli genotypes 1086 and high-glucoraphanin (HG). The start ATG codon is highlighted in green. Introns are highlighted in blue. Red boxes are polymorphisms that differentiate the *MYB28* allele in *B. villosa* from that in standard broccoli, including an AT microsatellite upstream of the start codon. The green box is the R2R3 DNA-binding domain. * indicates identical base in the four sequenced genomes.

The enhanced level of aliphatic methionine-derived glucosinolates in the high glucoraphanin genotypes is likely to be due to the enhanced expression of the *MYB28* C2 allele (Figure 3A). To provide further insight as to the possible cause of the enhanced expression, the CDS and 5′ upstream regions of *MYB28* C2 of *B. villosa*, HG Inbred, and 1086 were compared with that of the commercial broccoli, Ironman. Only one single nucleotide polymorphism (SNP) was found in the sequence coding of the R2R3 DNA-binding domain (Figure 4). This SNP is synonymous and was previously used to confirm the introgression of the *B. villosa* allele into the high glucoraphanin broccoli genotypes (Traka et al., 2013; Supplementary Figure 2). The most significant difference between the sequences was a polymorphism in an AT microsatellite or short tandem repeat (STR) upstream of the ATG start codon in which the length of the A/T sequence was longer in *B. villosa*, HG Inbred and 1086 compared to Ironman in which it was largely absent (Figure 4). To further explore this variable region, RNA-Seq data from *B. villosa* and the HG Inbred genotypes were mapped against TO1000. It was apparent that the AT-STR (AT STR) was situated within the 5′ untranslated region (5′UTR), and that the significant difference in sequence prevented the mapping of the broccoli and *B. villosa* sequence data to TO1000. It was also found that the AT STR was absent from the domesticated broccoli *B. oleracea* reference sequence DH1012, in a similar manner to Ironman (Chen et al., 2021).^4^

## Discussion

The use of wild relatives as a source of novel traits for crops has been widely advocated for several decades. There has been substantial investment in the collection and conservation of wild species both in seed banks and tissue culture facilities and *in situ* “gene parks.” However, the use of wild species has been limited, with the most prevalent use being for the introgression of “Mendelian” genes for disease resistance in crop cultivars. The wider use of wild species for other traits has been largely inhibited due to the extent of the backcrossing programme required to recover elite agronomic phenotypes. Advances in “genotype by sequencing approaches” provide significant opportunities for characterisation of the extent of introgression of both target and non-target genome sequences from alien species into crop genomes. We describe as an exemplar the quantification of both the extent of introgression of unique *B. villosa* sequences into an elite broccoli genetic background and the distribution of the introgressed fragments through a k-mer analysis (Figure 2 and Table 1). With the exception of the introgression of the *B. villosa MYB28* allele at the C2 locus that resulted in the enhanced levels of aliphatic glucosinolates, it was not evident that there were any other phenotypic consequences of agronomic importance due to the *B. villosa* introgressions. The extent of introgression of *B. villosa* was similar in the agronomically elite F_1_ hybrid 1086 and the inbred line HG Inbred, which lacks an agronomic phenotype. It is likely that the *MYB28* C2 introgression would have resulted in extensive changes to gene expression over and above that solely concerned with glucosinolate biosynthesis, even with no obvious phenotypic consequences, as it has previously been shown in *Arabidopsis* that *MYB28* regulates the expression of 240 genes of which only 13 were associated with glucosinolate biosynthesis (Hirai et al., 2007). It is also likely that other introgressions would have altered gene expression, although again with no obvious phenotypic effects.

In addition to the importance of *MYB28* in regulating aliphatic glucosinolates, the MYB29 transcription factor has also been associated with the regulation of aliphatic glucosinolate biosynthesis in *Brassica* (Zuluaga et al., 2019). There was not, however, a significant difference in expression of *MYB29* between Ironman and HG Inbred (Figure 3, *p* = 0.681).

Despite the success in identifying the extent of introgression of *B. villosa* into the high glucoraphanin genotypes, although it appeared to have no phenotypic effects apart from enhancing aliphatic glucosinolates, the precision of the analyses was significantly compromised by the availability of broccoli genotypes. Ironman was used as a surrogate for the actual genetic background of the high glucoraphanin genotypes. Likewise, the *B. villosa* genotype used in the current analyses is not the precise same genotype used in the original cross with cultivated *B. oleracea*. This may account for the difference in the length of the AT STR near the ATG start codon, as discussed below (Figure 4).

The genomic sequence of the *MYB28* C2 gene identified SNPs in both the coding and upstream non-CDS that confirmed the *MYB28* alleles in HG Inbred and 1086 were from *B. villosa* (Figure 4). The most extensive polymorphism between the *MYB28* C2 alleles was a variable AT microsatellite or STR 8 bases upstream from the ATG start codon which occurred in *B. villosa* and the high glucoraphanin introgression genotypes but was absent from the low glucosinolate genotype Ironman (Figure 4). It was found to be absent from the domesticated *B. oleracea* sequence DH1012. RNAseq analyses suggest that this AT STR was within the 5′-UTR as opposed to being within the promoter itself.

The role of polymorphisms in microsatellites or short tandems repeats in promoter regions that regulate gene expression and complex traits has been extensively explored in human genomics (Sawaya et al., 2013; Quilez et al., 2016; Fotsing et al., 2019) and has been associated with, for example, susceptibility to coronary heart disease (Chen et al., 2002). TATA boxes (ie a non cis regulatory coding sequence of repeating adenine and thymine bases) that promote transcription are a common element of the *Arabidopsis* genome (Molina and Grotewold, 2005), and genome-wide analyses have strongly associated variation in the length of STR with variation in gene expression in a similar manner to that more widely investigated in the human genome (Reinar et al., 2021). Moreover, STR polymorphic sequences in *Arabidopsis* that were associated with variable levels of gene expression have been shown to be clustered upstream of transcriptional start sites and especially of genes associated with response to biotic and abiotic stimuli (Reinar et al., 2021). Variation in the length of the STR may provide a means for both determining basal expression and fine tuning the response to external stimuli. Thus, it is conceivable that the variable AT STR that is upstream of the transcriptional start site of *MYB28* C2 in the broccoli genotypes is important in regulating the expression of *MYB28*, possibly by affecting the stability of the *MYB28* mRNA. This hypothesis needs to be further explored with additional sequencing of *Brassica* genotypes with variable levels of glucosinolates. The majority of wild forms of *B. oleracea* have high levels of methionine-derived glucosinolates with 2-propenyl, 3-butenyl side, 3methylthiopropyl, or 4-methylthiobutyl side chains that produce volatile and lachrymatory isothiocyanates, largely as a defence mechanism against generalist herbivores. Domestication of such wild *Brassica* may have led to the selection of genotypes that had reduced levels of these compounds possibly due to the selection of mutations that had a reduced length of the *MYB28* C2 STR that resulted in a reduced expression of *MYB28* and the suite of genes involved in glucosinolate biosynthesis.

## Data Availability Statement

The datasets presented in this study can be found in online repositories. The names of the repository/repositories and accession number(s) can be found below: National Center for Biotechnology Information (NCBI) BioProject database under accession number PRJNA623495.

## Author Contributions

MN undertook laboratory experimental studies, RNA-seq analysis, and field work. BS provided expertise in genome sequencing, K-mer analyses, and bioinformatics. PT-R and MHT provided expertise in RNA-seq analyses and gene ontology analyses. SS provided expertise and oversight for biochemical analyses. FB provided broccoli breeding genotypes and F_1_ hybrids and provided advice on broccoli breeding. MHT, LØ, and RM devised the research programme and provided oversight for all experimental work and analyses. MN, BS, and RM prepared the manuscript. All authors contributed to the article and approved the submitted version.

## Conflict of Interest

The broccoli with elevated glucoraphanin is the subject of patents filed by Plant Bioscience Limited (PBL), the technology transfer company of the John Innes Centre. RM and MHT are inventors named on these patents. FB was employed by Bayer. The remaining authors declare that the research was conducted in the absence of any commercial or financial relationships that could be construed as a potential conflict of interest.

## Publisher’s Note

All claims expressed in this article are solely those of the authors and do not necessarily represent those of their affiliated organizations, or those of the publisher, the editors and the reviewers. Any product that may be evaluated in this article, or claim that may be made by its manufacturer, is not guaranteed or endorsed by the publisher.

## Footnotes

1
https://en.novogene.com

2
https://digitalinsights.qiagen.com/

3
http://www.cbcb.umd.edu/software/jellyfish

4
http://brassicadb.cn/
