# Supplementary material for: Characterisation of the Introgression of Brassica villosa Genome Into Broccoli to Enhance Methionine-Derived Glucosinolates and Associated Health Benefits
Source: Front Plant Sci. 2022 Apr 1;13:855707. doi: 10.3389/fpls.2022.855707 (PMC9011106; doi:10.3389/fpls.2022.855707)
Supplement: Supplementary file 1 [file Data_Sheet_1.docx]

Neequaye et al. Supplementary Material

# Supplementary Table 1

| Sample | Species | Genotype | Total reads | Average depth(X) |
| --- | --- | --- | --- | --- |
| Ironman | *Brassica oleracea* | *MYB28^b/b^* | 122,472,326 | 33.23 |
| HG Inbred | *Brassica oleracea* | *MYB28^v/v^* | 130,769,980 | 36.14 |
| 1086 | *Brassica oleracea* | *MYB28^v/v^* | 122,086,950 | 34.87 |
| Brassica villosa | *Brassica villosa* | *MYB28^v/v^* | 127,819,586 | 36.31 |

Table of raw sequencing data for each sample including, sample name, species MYB28 genotype, Total number of effective reads in clean data and the average depth of reads at each site, calculated by the total number of bases in the reads divided by size of the assembled genome

# Supplementary Table 2

| Name | Species | Genotype | Total Length (bp) | Number of Contigs | Largest Contig (bp) | N50 (bp) |
| --- | --- | --- | --- | --- | --- | --- |
| Ironman | *Brassica oleracea* | *MYB28^b/b^* | 424718746 | 195078 | 83262 | 3840 |
| HG Inbred | *Brassica oleracea* | *MYB28^v/v^* | 427100513 | 166969 | 83262 | 5084 |
| 1086 | *Brassica oleracea* | *MYB28^v/v^* | 399827247 | 108592 | 86730 | 8041 |
| *Brassica villosa* | *Brassica villosa* | *MYB28^v/v^* | 396236782 | 136386 | 109455 | 6114 |

Table of assembly data including sample name, species, MYB28 genotype, total assembly length (bp), number of contigs making up the assembly, the length of the longest contig (bp) and the N50 which is when half of the total genome is covered by contigs longer than or equal to N50 (bp).

**Supplementary Table 3**

| *Gene of Interest* | *Primer Orientation* | Sequence |
| --- | --- | --- |
| *Housekeeping Gene UBC* | *Forward* | *GATCCACCCACCTCGTGTAG* |
| *Housekeeping Gene UBC* | *Reverse* | *CTGGAGGGAAGTGAATGGTAAC* |
| *MAM C7* (Bo7g098000)* | *Forward* | *ACCGCAGAAGCTTGAGATTG* |
| *MAM C7* (Bo7g098000)* | *Reverse* | *CTTCATCCACCTCATTTCCC* |
| *CYP79F1/2 (Bo5g021810)* | *Forward* | *GACGAGAGGGTGGAGTTATG* |
| *CYP79F1/2 (Bo5g021810)* | *Reverse* | *AATTCAACGCATTGAGCTTT* |
| *APS3 (Bo1g057690)* | *Forward* | *CAATTCGCTTCGTCTCGACG* |
| *APS3 (Bo1g057690)* | *Reverse* | *CTCAATGTCGCTGAGAATAGCG* |

RT-qPCR Primers used to quantify expression of genes found to be significantly differentially expressed in the RNAseq analysis. This gene has previously be assigned MAM3 (Yin et al. 2017)

**Supplementary Table 4**

ANOVA of total aliphatic glucosinolate content in florets of four broccoli genotypes with Tukey’s multiple comparison tests

**Supplementary Figure 1**


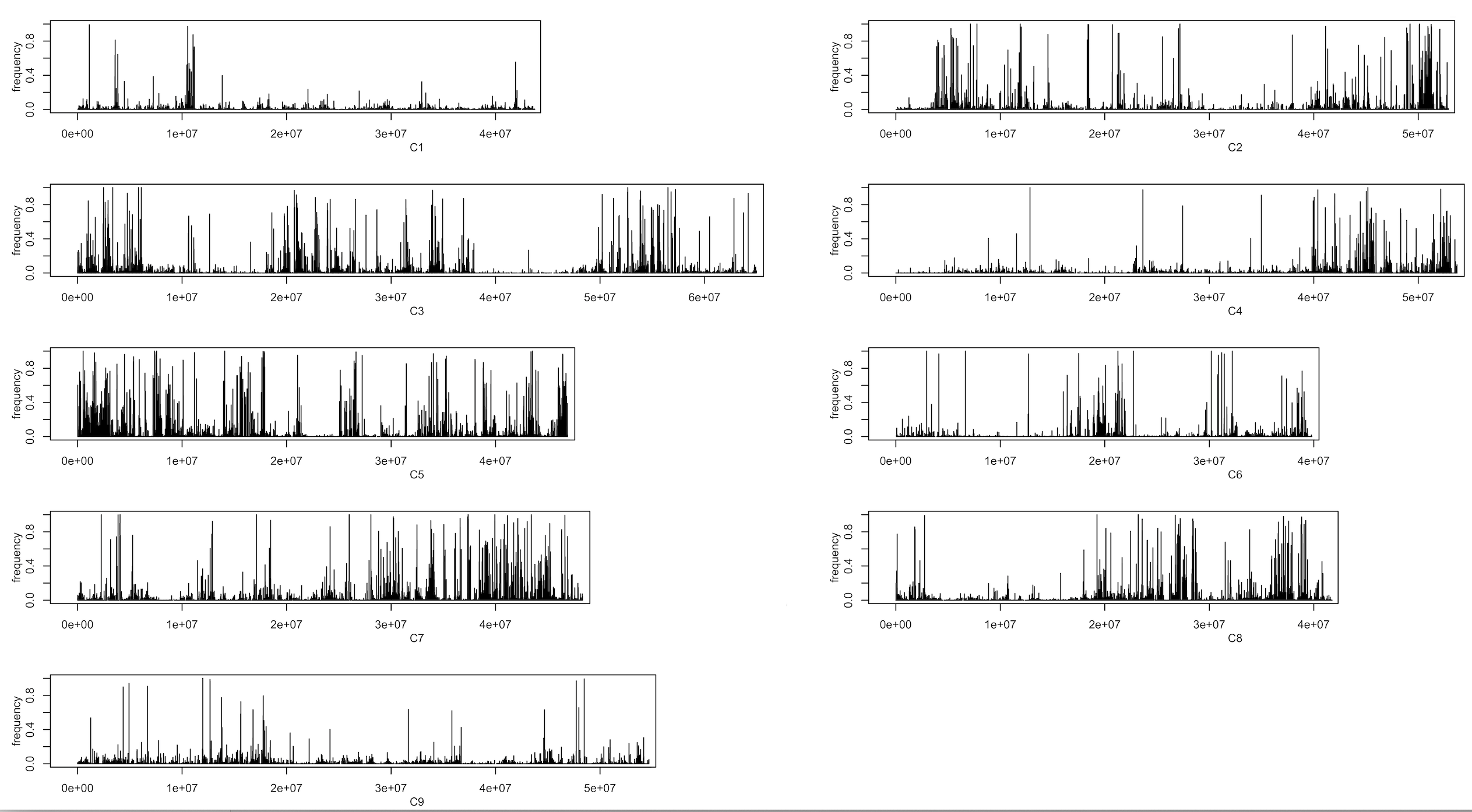


Signals for potential introgression traces from *B.villosa* Introgression from K-mer analyses in the HG Inbred. Each black horizontal line in the plot marks a contig of a draft-assembled high-glucoraphanin genome. The height of each line is according to the fraction of kmers in a contig that is unique to *B. villosa*.

# Supplementary Figure 2


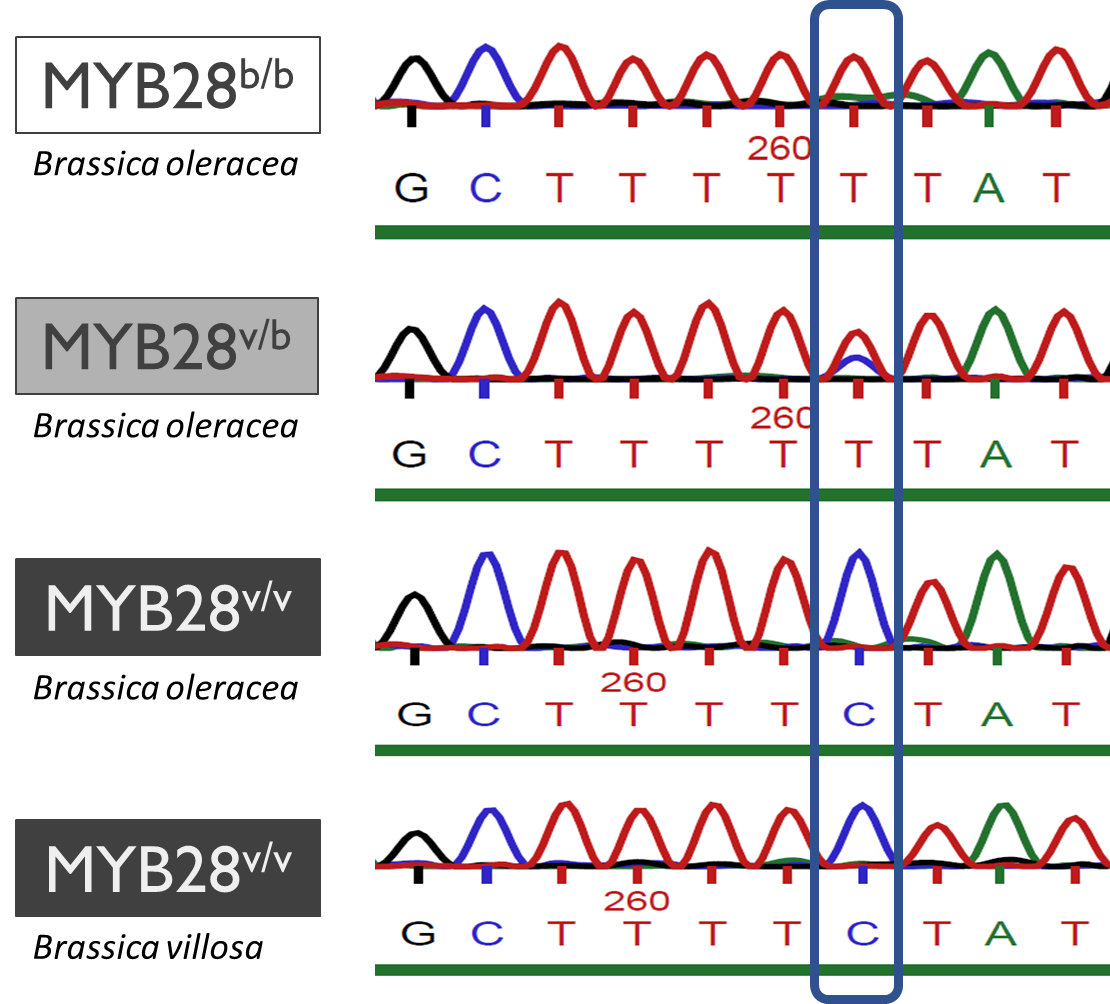


*The diagnostic SNP used to determine MYB28 genotypes of the two HG broccoli cultivars, 1199 MYB28^v/b^,and HG Inbred MYB28^v/v^, derived from crossing of standard broccoli MYB28^b/b^(top) and B. villosa MYB28^v/v^ (bottom)*

Yin L, Chen H, Cao B, Lei J, Chen G (2017) Molecular Characterization of MYB28 Involved in Aliphatic Glucosinolate Biosynthesis in Chinese Kale (Brassica oleracea var. alboglabra Bailey). Front Plant Sci 8:1083
